# Potentially inappropriate medication use among hypertensive older African-American adults

**DOI:** 10.1186/s12877-018-0926-9

**Published:** 2018-10-05

**Authors:** Mohsen Bazargan, James L Smith, Ebony O King

**Affiliations:** 1 Charles R. Drew University of Medicine and Science, 1731 East 120th Street, Los Angeles, CA 90005 USA; 2 University of California, Los Angeles, USA

**Keywords:** Potentially inappropriate medication (PIM), Minority, Older adults, Pain, Falls

## Abstract

**Background:**

Inappropriate use of medications, particularly among minority older adults with co-morbidity, remains a major public health concern. The American Geriatrics Society (AGS) reports that Potentially Inappropriate Medication (PIM) continues to be prescribed for older adults, despite evidence of poor outcomes. The main objective of this study was to examine the prevalence of PIM use among underserved non-institutionalized hypertensive older African-American adults. Furthermore, this study examines potential correlations between PIM use and the number and type of chronic conditions.

**Methods:**

This cross-sectional study is comprised of a convenience sample of 193 hypertensive non-institutionalized African-American adults, aged 65 years and older recruited from several senior housing units located in underserved areas of South Los Angeles. The updated 2015 AGS Beers Criteria was used to identify participants using PIMs.

**Results:**

Almost one out of two participants had inappropriate medication use. While the average number of PIMs taken was 0.87 drugs, the range was from one to seven medications. Almost 23% of PIMs were due to drugs with potential drug-drug interactions. The most common PIM was the use of proton pump inhibitors (PPI) and Central Nervous System (CNS) active agents. Nearly 56% of PIMs potentially increased the risk of falls and fall-associated bone fractures. The use of PIMs was significantly higher among participants who reported a higher number of chronic conditions. Nearly 70% of participants with PIM use reported suffering from chronic pain.

**Conclusions:**

The major reason for high levels of polypharmacy, PIMs, and drug interactions is that patients suffer from multiple chronic conditions. But it may not be possible or necessary to treat all chronic conditions. Therefore, the goals of care should be explicitly reviewed with the patient in order to determine which of the many chronic conditions has the greatest impact on the life goals and/or functional priorities of the patient. Those drugs that have a limited impact on the patient’s functional priorities and that may cause harmful drug-drug interactions can be reduced or eliminated, while the remaining medications can focus on the most important functional priorities of the patient.

## Background

Older African-American adults have a high prevalence of many of the most potent cardiovascular disease risk factors, particularly hypertension (HTN) and diabetes mellitus [1]. The combination of HTN and diabetes is lethal and is associated with elevated all-cause and cardiovascular disease mortality among older adults [2, 3]. In 2015, more than 71% of older African American Medicare fee-for-service beneficiaries had at least two chronic conditions and 43% suffered from at least four conditions [4]. National data show that 39.8%, 45.6%, and 64.4% of older African-American Medicare adult beneficiaries have diabetes, hyperlipidemia, and HTN, respectively [5]. Effective treatment of these conditions often requires the prescribing of multiple medications. Within the last decade, prescription medication (Rx) use has increased dramatically among older adults, i.e., the median number of Rx used doubled from 2 to 4, and those taking more than 5 medications tripled from 13 to 39% [6]. This increase in medication use among older adults was driven, in part, by higher use of antidiabetics, anti-hypertensives, and statins [6]. The use of these medications has led to substantial improvements in Low-Density Lipoprotein Cholesterol, blood pressure and glycemic control. Despite these favorable outcomes, suboptimal use of medications among ethnic/racial population limits maximal health benefits [7–9]. Three major medication-related challenges that lead to suboptimal use of medications are: 1) nonadherence to prescribed drug regimens [10–16], 2) excessive and unnecessary use of medication [17, 18], and 3) inappropriate use of medications [19].

The American Geriatrics Society reports that Potentially Inappropriate Medication (PIM) continues to be prescribed for older adults, despite evidence of poor outcomes from the use of PIMs in this segment of our population [20]. Several studies documented that PIM use has been associated with an increased risk of falls and hip fractures [21]. A recent systematic review documented that PIM use was associated with a 1.6-fold increased mortality in older adults [22]. Another recent meta-analysis of PIM use in community-dwelling older American adults found the median rate of PIM to be 19.6% with a range of 4.5 to 33.3% [23].

Prevalence estimates for PIMs represent an important healthcare quality metric [24]. Specifically, given the high rate of PIM use among underserved older African-American adults, there is a need for ongoing monitoring of inappropriate medication use among this segment of our population. Additionally, it is important to continue investigating the extent and consequences of PIM use among those older African-American adults with medical conditions, such as cardiovascular disease, that place them at risk for poorer clinical outcomes. A recent study that examined PIM use among a large sample (*n* = 16,588) of non-institutionalized older adults (age ≥ 65) from the 2006–2010 Medical Expenditure Panel Survey documented strong associations between PIM use and cardiovascular disease and other chronic conditions [24].

While several studies in the United States and other countries have examined the prevalence and correlates of PIM use among aged populations [23, 25–32], to the best of our knowledge, few studies have specifically examined the older African-American community [19]. The main objective of this study is to examine PIM use among noninstitutionalized hypertensive older African-American adults, using the revised Beers’ criteria (2015), to identify those individuals that may be disproportionately affected by inappropriate drug use to be targeted for appropriate interventions to reduce the use of PIMs. Furthermore, this study examines potential correlations between PIM use and the number and type of chronic conditions. In addition, we present five cases in order to gain insight into PIM use among our sample. These case studies provide a variety of actual scenarios that may help providers to reexamine PIM issues from their perspectives and then synthesize a solution in their own practice.

## Methods

This cross-sectional observational study is comprised of a convenience sample of 193 non-institutionalized African-American adults, aged 65 years and older, with a clinical diagnosis of hypertension.

### Participants and setting

Participants were recruited from several predominantly African-American senior centers and housing units located in underserved areas of South Los Angeles. This study is part of a larger effort to examine medication use among a sample of 342 African Americans aged 55 years and older. However, the current study used only participants that were 65 years of age or older (193) from the study sample to examine the use of PIMs. Only four potential subjects refused to participate in our study. No participants were selected that were residing in skilled nursing facilities. Potential participants were excluded if they were enrolled in other clinical trials. In addition, using a standard screening tool, potential participants with cognitive deficits were excluded from the study. This investigation was approved by the Charles R. Drew University of Medicine and Science Institutional Review Board. A written informed consent was collected from all participants. The study used structured, face-to-face survey interviews. Data used in the study were collected from November 2015 – February 2017.

#### Measurement

The survey instrument was a collection of validated instruments from various sources [11, 17, 19, 33–35]. Demographic variables included age, gender, education, living arrangement, and disability status.

*Medication use* was assessed using the drug inventory method. Participants were asked to bring all over-the-counter and Rx medications that were taken within 2 weeks prior to the interviews. The interviewer transcribed from the container label the name of the medication, strength of the drug, expiration date, instructions, special warnings, providers’ information, etc. The medication assessment of this study employed the methodology established by Sorensen and colleagues [36–38], which was adopted by our research team previously [11, 17, 19, 33–35].

### PIM use

The updated 2015 AGS Beers Criteria was used to identify PIM [20]. In this study PIM was defined as the number of medications that must be avoided but were prescribed for an older adult. Based on the 2015 AGS Beers Criteria, calculation of PIM should not count a prescription as a PIM in the presence of certain indications due to specific clinical conditions and criteria [Examples: use of proton-pump inhibitors (PPI) for less than 8 weeks; use of antipsychotics, first- (conventional) and second- (atypical) generation for treatment of schizophrenia, bipolar disorder, or short-term use as antiemetic during chemotherapy; use of non-cyclooxygenase-NSAIDs, oral for young-old (< 75 years) for short duration; etc.] [20]. In our study, a prescribed medication was considered appropriate (not being counted as a PIM) using the above Beers criterion in 19 PIMs (17 participants).

The AGS 2015 Updated Beers Criteria lists the drug-drug and drug-disease interactions that should be avoided in older adults. We documented the presence of these interactions by referencing this list. PIMs were counted as a total number and an adjusted number, the latter indicating a likely elimination of a medication as a PIM if a medical exception was met.

### Chronic conditions

Participants were asked to report on diagnoses only if a physician had confirmed them previously. We collected information about 13 conditions: diabetes, hypertension, thyroid disorder, cardiac disease, cancer, asthma, osteoarthritis, rheumatoid arthritis, chronic obstructive pulmonary disease, intestinal disease, depression, and hypercholesterolemia. We also used an alternative method based on the labels of medication containers to identify participants’ chronic conditions. We assigned a condition to a participant if the therapeutic purpose of the medications used was clearly linked to the presence of a chronic condition. This method (examination of the medication containers) leads to an additional 25 conditions. However, a vast majority of these 25 medical conditions was not frequently diagnosed, including lupus, hepatitis, vertigo, cachexia, herpes, edema, seizures etc. Relying on self-report or administrative data alone in documenting chronic conditions underestimates the prevalence of chronic conditions, which results in biased estimates of multi-morbidity [39].

### Sample size and statistical analysis

Both descriptive and inferential statistics were used to document prevalence and correlates of PIM use among our sample. Pearson correlation coefficients, chi-squared tests, and the binary logistic regression techniques were used to examine the correlation between the PIM use and 1) number of medication use; 2) number of chronic conditions; and 3) type of chronic conditions. Examining correlation between several types of chronic conditions and PIM, the Bonferroni correction was used to counteract the problem of inflated type I errors. All statistical analyses were performed with the Statistical Package for Social Sciences (SPSS) version 22. Based on previous studies, we expected at least 30% of study participants will be using at least one PIM [19, 23]. Therefore the sample size of 193 subjects is sufficient to determine the extent of PIM use among older African American adults.

## Results

Table 1 reports the demographic characteristics and health status of our sample. There were 193 study participants, with an age range from 65 to 96 years (M 75.2 ± 7). Approximately 48% of the participants were 75 years of age or older and 67% of the participants were women. The number of reported chronic illnesses ranged from one to 17, with the average being eight (7.8 ± 3.2). One out of four participants had at least 10 chronic conditions. Participants were taking an average of 7.3 (SD = 3.60) prescription drugs (range: 1–24). Thirty percent of participants were using at least nine medications, whereas 27% and 43% of participants were taking five to eight and one to four prescription medications, respectively (Table 1). In addition, our data shows that nearly 21%, 36%, 60%, 65% of participants were diagnosed with depression, diabetes mellitus, hyperlipidemia, and chronic pain, respectively.

**Table 1:** Demographic characteristics and health status of sample (*N* = 193)

Demographic and health status	N (%)
Age	
65–74 years	100 (52)
≥ 75 years	93 (48)
Gender	
Male	63 (33)
Female	130 (67)
Education	
No high school diploma	48 (25)
High school diploma	68 (35)
Some college or more	77 (40)
Living Arrangement	
Living alone	150 (78)
Living with someone	42 (22)
Disability Status	
Yes	89 (44)
No	104 (54)
Diabetes	
Yes	35 (18)
No	158 (82)
Hyperlipidemia	
Yes	115 (60)
No	78 (40)
Chronic Pain	
Yes	125 (65)
No	68 (35)
Depression	
Yes	41 (21)
No	152 (79)
Number of Prescribed Medications	
0–4	52 (27)
5–8	84 (43)
9–24	57 (30)
Number of Chronic Conditions	
1–5	55 (29)
6–10	93 (48)
11–17	45 (23)

Our data indicates that inappropriate drug use occurred in 46% (99) of participants. In addition, 26% (55) 13% (25) and 8% (19) of participants were taking one, two or at least three inappropriate medications, respectively. While the average number of PIMs taken was 0.97 drugs, the range was from one to seven medications. A total of 188 PIMs were used by ninety-nine individuals. Almost 23% (43 out of 188 PIMs) were due to drugs with potential drug-drug interactions. The most common PIM was the use of proton pump inhibitors (PPI) and greater than two or more Central Nervous System (CNS) active agents, occurring at 46% and 18%, respectively. Nearly 56% (105 out of 188) of PIMs potentially increased the risk of falls and fall-associated bone fractures.

The use of PIMs was significantly higher among participants who were taking a higher number of medications (*r* = 0.51; *p* < .0001). Participants who were taking at least six medications were 3.6 times (OR = 3.6; CI: 1.91–6.7; *p* < 0.001) more likely to also be receiving inappropriate medications. Similarly, the use of PIMs was significantly higher among participants who reported a higher number of chronic conditions (*r* = 0.46; *p* < 0.0001). Participants who were diagnosed with at least six chronic conditions were 2.7 times (OR = 2.73; CI: 1.40–5.4; *p* < 0.001) more likely to be receiving inappropriate medications. In addition, three chronic medical conditions showed a statistically significant independent correlation with PIM use. Participants who were diagnosed with chronic pain (OR = 2.4; CI: 1.3–4.5; *P* < .005), depression (OR = 2.8; CI: 1.4–5.8; *P* < .005), and gastroesophageal reflux disease (OR = 2.9; CI: 1.6–5.3; *P* < .0001) were 2.4, 2.8 and 2.9 times, respectively, more likely to use PIMs than their counterparts without these chronic conditions.

### Case studies

#### Case 47

The participant is in her 70s with a medical history of osteoporosis, recent fall (12 months ago), depression, sleep disorder, and chronic pain. She has a total of 12 self-reported medical conditions and is taking a total of five potentially inappropriate medications (PIMs). At the time of the survey, the participant was taking a PPI for 12 months. PPI use longer than 8 weeks is considered a PIM as it increases the risk of developing *Clostridium difficile* infection and bone loss and fractures. She was also taking nortriptyline, a tricyclic anti-depressant with strong anti-cholinergic properties, and hydrocodone-acetaminophen. These two drugs separately and together have the potential of a > 2 CNS active agent drug-drug interaction, increasing the risk of falls and/or fractures. Lastly, the participant is taking meloxicam, a non-steroidal anti-inflammatory drug (NSAID), which in patients older than 74 years of age, poses considerable risk for peptic ulcer disease and/or development of gastrointestinal bleeding.

#### Case 102

The participant is in her 70’s with a medical history significant for Parkinson’s disease, dependent activities of daily living (ADLs), depression and heart problems with a pacemaker. She has a total of 12 self-reported medical conditions, one-third of which are associated with PIMs that pose a significant risk of morbidity. At the time of the survey, the participant was taking a PPI for 2 weeks, although the medication had been prescribed for 12 months. She was also taking hydrocodone-acetaminophen and risperidone, which together has the potential of a > 2 CNS active agent drug-drug interaction increasing the risk of falls and/or fractures. In the case of this participant, this would hold true as the participant has both dependent ADLs and Parkinson’s disease. Of particular significance is the use of the anti-psychotic risperidone in this participant with her concurrent diagnoses of Parkinson’s disease. All anti-psychotics, with the exception of aripiprazole, quetiapine, and clozapine, are believed to carry the risk of the precipitation and worsening of Parkinson’s symptoms.

#### Case 229

The participant is in her 80s with a medical history significant for hypertension, sleep disorder and chronic pain. She has a total of eight self-reported medical conditions and a total of six PIMs including three separate drug-drug interactions. The participant is taking triamterene and lisinopril which together pose a risk for drug-drug interactions due to an increased risk of hyperkalemia. The participant is also taking temazapam, which by itself and together with nortriptyline and hydrocodone poses two separate > 2 CNS drug interactions and increases the risk of cognitive impairment, delirium, falls, fractures, and motor-vehicle accidents. Nortriptyline by itself and together with hydrocodone and temazapam has the potential of two separate > 2 CNS drug-drug interactions and present a PIM with risks as described above. The participant has a significant risk for morbidity for taking > 3 CNS active agents.

#### Case 249

The participant is in his seventies with a medical history significant for dependent ADLs, heart problems, chronic pain and a sleep disorder. He has a total of 13 self-reported medical conditions and is taking a total of 13 medications, six of which are PIMs including three sets of drug-drug interactions. Tramadol and codeine are both opiates and are identified as PIMs as individual drugs in patients with a history or risk of falls or fracture, as is the case in this patient with dependent ADLs. Together these drugs also pose a > 2 CNS active drug-drug interaction. The participant is also taking a corticosteroid and NSAIDs, which together can cause a drug-drug interaction and increase the risk of peptic ulcer disease and/or gastrointestinal bleeding. Lastly, the participant is taking diphenhydramine, which by itself is a PIM in patients with a history or risks of falls and/or fractures. Diphenhydramine together with solifenacin is a risk for drug-drug interaction as both drugs are strong anticholinergics, increasing the risk of cognitive decline.

#### Case 288

The participant is in her 80s with a medical history of dependent ADLs, rheumatoid arthritis, thyroid disease, and breast cancer remission. She also has a total of 16 self-reported medical conditions, is taking a total of 16 medications, seven of which are PIMs including three sets of drug-drug interactions. The participant is taking lorazepam, oxycodone, and morphine, which by themselves are PIM in patients with a history or risk of falls and/or fracture, as is the case of this participant who has dependent ADLs. Together these CNS active agents create three different sets of > 2 CNS active agent drug-drug interactions. Lastly, the participant was taking a PPI for 7 months, which increases the risk of clostridium difficile, fractures, and falls.

## Discussion

Use of medication that may cause more harm than benefit is considered potentially inappropriate when safer pharmacological or non-pharmacological alternatives exist [40]. Older African-American adults are a particularly vulnerable population as they suffer from a higher number of chronic conditions than their white counterparts. Our data show that 41% of older hypertensive African Americans were taking at least eight medications. In addition, one out of four participants reported at least 10 chronic conditions. Yet we documented that one out of two participants are taking at least one inappropriate medication. Similar to this study, a recent study conducted among a sample of 400 older underserved African-American adults reported a high rate of PIM use [19]. The rate of PIM use in our study is substantially higher than previously reported PIM use among older adults. The 2006–2010 Medical Expenditure Panel Survey (MEPS), shows that one-third (32.8%) of older African-American adults used a PIM [24]. The higher rate of PIM use in our sample may reflect the fact that our sample has been recruited from the Service Planning Area 6 (SPA 6) of Los Angeles County, one of the most underserved minority communities in the US. We specifically selected SPA 6 because nearly half of older adults living in SPA 6 are African American (49%). Home to more than one million residents, SPA 6 has disproportionately greater health disparities than the rest of Los Angeles County [41]. A good example is the age-adjusted coronary heart disease death rate which is significantly higher for SPA 6 (147.5 per 100,000 population) than the Nation (102.6) and the rest of Los Angeles County (116.7). Another example is the aged-adjusted diabetes death rate which is 37.6, 21.9, and 21.2 per 100,000 population for the SPA 6, Los Angeles County and the Nation, respectively [41].

Both quantitative analysis and the cases reported here show that chronic pain is one of the important common denominators of PIM use. Fifty-four percent of our study population who were diagnosed with chronic pain consume at least one PIM. Indeed, it is not surprising that suffering from chronic pain leads to PIM use. Previous studies have also shown that inappropriate pain medications are frequently prescribed for older adults [42]. However, empirical evidence suggests that older African Americans have a higher risk for severe pain compared with non-Hispanic whites [43]. Yet severe mismanagement of pain in underserved older African Americans, particularly those with comorbidity, multiple providers, and limited access to health care has been documented [19]. A recent study shows that one in four older African American were taking NSAIDs, which can cause serious side effects in older adults with multiple chronic conditions [19]. The use of pain medication was associated with drug-drug interactions, drug duplication, and PIM use [19]. These results support the need for clinicians to be aware of PIM use by older adults, recognize associated medication-related adverse events, and avoid prescribing age-inappropriate medications to vulnerable older adult patients [42].

Another commonly prescribed PIM in our study was taken by participants who were diagnosed with the gastroesophageal reflux disease (GERD). A recent study examining the PIM use among older patients with cardiovascular disease also listed the unnecessary use of PPIs common among older adults [44]. It is important that providers carefully consider the use of PPIs in older adults and monitor their continued use to prevent the drug-drug interactions and side effects, including risk of *Clostridium difficile* infection, bone loss, and fractures [45].

In addition, another common denominator for PIM use, both in our case studies and in the quantitative analysis, was diagnoses of depression among our older African-American adults. Given that several psychotropic drugs are included in Beers’ list, it was not surprising to find that the presence of depressive symptoms was associated with potentially inappropriate medication use. Other studies have confirmed this association [46]. It has been empirically documented that use of anti-cholinergic medications that are listed as PIMs leads to cognitive declines and dementia among older adults [47]. Therefore, it is important that physicians avoid prescribing anti-cholinergic medication listed as PIMs, particularly for old-old adults [47]. In addition, the standard care for dementia should include careful medication review and management to avoid PIM use in this vulnerable population [48].

Another overarching theme that emerged from our case reports is the history of falls and use of PIMs that substantially increases the risk of falls among our participants. Our data shows that 56% of PIMs used by our participants potentially increased the risk of falls and fall-associated bone fractures. Several studies documented that PIM use has been associated with an increased risk of falls and hip fractures [21]. There are mixed findings in the literature in regards to the racial differences on falls among older adults. Many studies found that older non-Hispanic Whites were more likely to fall than older African Americans [49–51], yet several studies revealed no racial differences or excessive falls rates among African-American older adults compared to their White counterparts [52–55]. However, African Americans have a greater fall-risk profile when compared to non-Hispanic Whites. Older African Americans’ higher fall-risk scores are attributed to their physical functioning and medication use [56] and poorer self-rated health and multiple comorbidities [57]. The 2014 Behavioral Risk Factor Surveillance System survey shows that regardless of ethnic background, the rate of fall-related injuries is significantly higher in the older adults with poor health (480 per 1000) than their counterparts with excellent health (69 per 1000) [58]. Managing at least one chronic disease increases the risk of falling by 32% [59]. The most recent report from the Center for Medicare and Medicaid Services (CMS) Chronic Condition Data Warehouse shows a higher prevalence of major chronic conditions among African-American older adults compared to their non-Hispanic White counterparts. African-American older adults experience higher rates of hypertension (68.4% vs 57.7), diabetes (39.8% vs. 24.7), chronic kidney disease (26.7 vs. 18.2), heart failure (17.9% vs. 14.3%), Alzheimer’s disease/dementia (14.2% vs. 11.3%) stroke (5.7% vs. 4.2) and cancer (10.1% vs. 9.1%). Therefore, it is imperative that providers who are prescribing medications to African-American older adults with multiple chronic conditions, be cognizant of the vulnerability of their patients and avoid prescribing the PIMs that increase the risk of falls.

### Limitations of the study

The research team did not have access to the participants’ medical records to record the medications that were prescribed for participants. Medication-related information was collected directly from drug containers. Second, the study used a convenience sample, which limits the generalizability of our findings. Finally, we used a cross-sectional study design, which allowed us to collect data at only a single point in time. Nevertheless, this study provides vital information about a population (underserved African-American older adults) that has not been carefully studied to this point.

## Conclusions

The revised AGS Beers criteria provides valuable tools to guide prescribing in older adults. All clinicians who provide medical care for older adults should be familiar with this tool. However, it is important to note that reducing PIMs is only one component of improving quality of care for older adults [60]. Relying only on Beers criteria obscures the detection of important drug-related problems such as drug use without an indication, untreated conditions, or poor adherence [61]. PIM, polypharmacy, and nonadherence to medications are all interconnected, and these factors are linked to effectiveness of medications and health outcomes among older adults [11, 19, 62]. However, no intervention trials have focused on these medication-related challenges together to improve adherence to medications among African-American older adults [63]. Therefore, the first step in reducing medication-related challenges and improving the effectiveness and management of medications among older adults, particularly older African-American adults, is to conduct a comprehensive assessment of their medications [64].

The likely reason for high levels of polypharmacy, PIMs, and drug interactions is that patients suffer from multiple chronic conditions. But it may not be possible or necessary to treat all chronic conditions. Therefore, the goals of care should be explicitly reviewed with the patient (or, if the patient has limited capacity for decision making, with the patient’s caretaker) in order to determine which of the many chronic conditions has the greatest impact on the life goals and/or functional priorities of the patient. Those drugs that have a limited impact on the patient’s functional priorities and that may cause harmful drug-drug interactions can be reduced or eliminated, while the remaining medications can focus on the most important functional priorities of the patient.

A recent interventional study used electronic alerts at the point of computerized order-entry to reduce PIM prescribing among a large sample of older veteran adults. The study showed a modest reduction in the rate of the top 10 most common newly-prescribed PIMs (from 9.0 to 8.3%; *p* = 0.016). This intervention was only able to modify the prescribing behaviors of neurologists and detected no positive impact on prescribing of other provider specialists [65]. This is another indication that multidimensional and multicomponent interventions are needed to focus on the relationship between comorbidities and medication-related challenges among African-American older adults [66, 67].

The four case reports given above clearly show that there is additional need for multidisciplinary interventions to reduce medication-related challenges among older African-American adults with comorbidities, particularly those who suffer from pain. Safe and appropriate prescribing is a complex process, involving issues of over-prescription, under-prescription, and inappropriate prescription [68]. Without close and continuous collaboration between providers and patients with a coherent multi-pronged strategy, de-prescribing, as part of the solution to address over-prescribing and inappropriate prescribing, is almost impossible.

## Abbreviations

ADL: Activities of daily living
AGS: American Geriatrics Society
CI: Confidence intervals
CMS: Center for Medicare and Medicaid Services
CNS: Central nervous system
GERD: Gastroesophageal reflux disease
HTN: Hypertension
MEPS: Medical expenditure panel survey
NSAID: Non-steroidal anti-inflammatory drug
OR: Odds ratio
PIM: Potentially inappropriate medication
PPI: Proton pump inhibitors
Rx: Prescription medication
SPA 6: Service planning area 6
SPSS: Statistical package for social sciences
